# On the Relation of Medicine to Surgery, and on the Threefold Division of the Medical Profession

**Published:** 1843-10

**Authors:** 


ART. III.—*Ueber das Verhältniss der Medicin zur Chirurgie, und die Dreiheit im heilenden Staate, &c.* Von Dr. C. H. BISCHOFF, &c.—*Bonn*, 1842. 8vo, pp. 102.

*On the Relation of Medicine to Surgery, and on the threefold Division of the Medical Profession.* By Dr. C. H. BISCHOFF, Royal Prussian State Councillor, Professor of Materia Medica, and of Military and Civil Medical Police, &c. in the University of Berlin.—*Bonn*, 1842.

THE excitement now prevalent in Germany respecting the constitution and internal organization of the medical profession is the more worthy of notice as being contemporary with a similar movement in this country. We have already noticed the state of the profession in Germany, in our review of Professor V. Walther's pamphlet, (Br. and For. Med. Review, vol. XIV. p. 402,) and we have little to add to the remarks we then made. Dr. Bischoff advocates a ternary division, namely, into the two classes of physicians and surgeons, equally educated in every respect, but distinct in practice; and a third class, comprising the assistants to the superior grades. Dr. Bischoff's arguments are mainly directed against the practice, now prevalent in Germany, of educating medico-chirurgical or general practitioners of an inferior grade of attainments, and have consequently a polemical tone neither pleasing nor convincing to the unprejudiced reader. It appears that the Medico-Chirurgs are poaching sadly upon the domains of the regularly-educated and titled doctors. Dr. Bischoff says that they argue thus with the public: "Herr A. is an excellent physician; Herr B. an excellent surgeon; and Herr C. is both: *ergo, fiat applicatio.*" And this argument is, and ever will be, valid and weighty. The public wants men who are both, as we have previously shown, and all the rules and regulations that can be devised will never prevent that want of the public being supplied. Medicine and surgery are one and indivisible in teaching or in practice. The greatest difficulty reformers have to encounter is, as to who shall dispense medicines and perform the less honorable and more mechanical duties of the surgeon. If they be left in the hands of the druggist, that useful individual becomes *ipso facto* a surgical physician of an inferior grade, and we are where we were, with a tribe of ignorant *sage femmes en culottes* into the bargain. Probably the wisest plan would be to intrust the inferior and less responsible duties to junior practitioners; making them, in fact, serve a sort of medical curacy for a title. A service of this kind, if compulsory, would come to be considered no more degrading than the *status pupillaris* of the medical student is now.

